# Heterogeneity of outcomes within diabetic patients with atrial fibrillation on edoxaban: a sub-analysis from the ETNA-AF Europe registry

**DOI:** 10.1007/s00392-022-02080-5

**Published:** 2022-08-17

**Authors:** Giuseppe Patti, Ladislav Pecen, Giuseppina Casalnuovo, Marius Constantin Manu, Paulus Kirchhof, Raffaele De Caterina

**Affiliations:** 1 Department of Translational Medicine, Maggiore della Carità Hospital, University of Eastern Piedmont, Via Solaroli 17, 28100 Novara, Italy; 2 Institute of Computer Science of the Czech Academy of Sciences, Prague, Czech Republic; 3 Medical Faculty, Charles University, Pilsen, Czech Republic; 4 Daiichi Sankyo Europe, Munich, Germany; 5 Department of Cardiology, University Heart and Vascular Centre UKE, Hamburg, Germany; 6 Institute of Cardiovascular Sciences, University of Birmingham, SWBH and UHB NHS Trusts, Birmingham, UK; 7 The Atrial Fibrillation NETwork (AFNET, Münster, Germany; 8 University Cardiology Division, Cardiovascular and Thoracic Department, Pisa University Hospital, University of Pisa, Via Paradisa, 2, 56124 Pisa, Italy

**Keywords:** Diabetes, Insulin, Atrial fibrillation, Thromboembolic events, Mortality

## Abstract

**Background:**

Recent data have suggested that insulin-requiring diabetes mostly contributes to the overall increase of thromboembolic risk in patients with atrial fibrillation (AF) on warfarin. We evaluated the prognostic role of a different diabetes status on clinical outcome in a large cohort of AF patients treated with edoxaban.

**Methods:**

We accessed individual patients’ data from the prospective, multicenter, ETNA-AF Europe Registry. We compared the rates of ischemic stroke/transient ischemic attack (TIA)/systemic embolism, myocardial infarction (MI), major bleeding and all-cause death at 2 years according to diabetes status.

**Results:**

Out of an overall population of 13,133 patients, 2885 had diabetes (22.0%), 605 of whom (21.0%) were on insulin. The yearly incidence of ischemic stroke/TIA/systemic embolism was 0.86% in patients without diabetes, 0.87% in diabetic patients not receiving insulin (*p* = 0.92 vs no diabetes) and 1.81% in those on insulin (*p* = 0.002 vs no diabetes; p = 0.014 vs diabetes not on insulin). The annual rates of MI and major bleeding were 0.40%, 0.43%, 1.04% and 0.90%, 1.10% and 1.71%, respectively. All-cause yearly mortality was 3.36%, 5.02% and 8.91%. At multivariate analysis, diabetes on insulin was associated with a higher rate of ischemic stroke/TIA/systemic embolism [adjusted HR 2.20, 95% CI 1.37–3.54, *p* = 0.0011 vs no diabetes + diabetes not on insulin] and all-cause death [aHR 2.13 (95% CI 1.68–2.68, *p* < 0.0001 vs no diabetes]. Diabetic patients not on insulin had a higher mortality [aHR 1.32 (1.11–1.57), *p* = 0.0015], but similar incidence of stroke/TIA/systemic embolism, MI and major bleeding, vs those without diabetes.

**Conclusions:**

In a real-world cohort of AF patients on edoxaban, diabetes requiring insulin therapy, rather than the presence of diabetes per se, appears to be an independent factor affecting the occurrence of thromboembolic events during follow-up. Regardless of the diabetes type, diabetic patients had a lower survival compared with those without diabetes.

**Graphical abstract:**

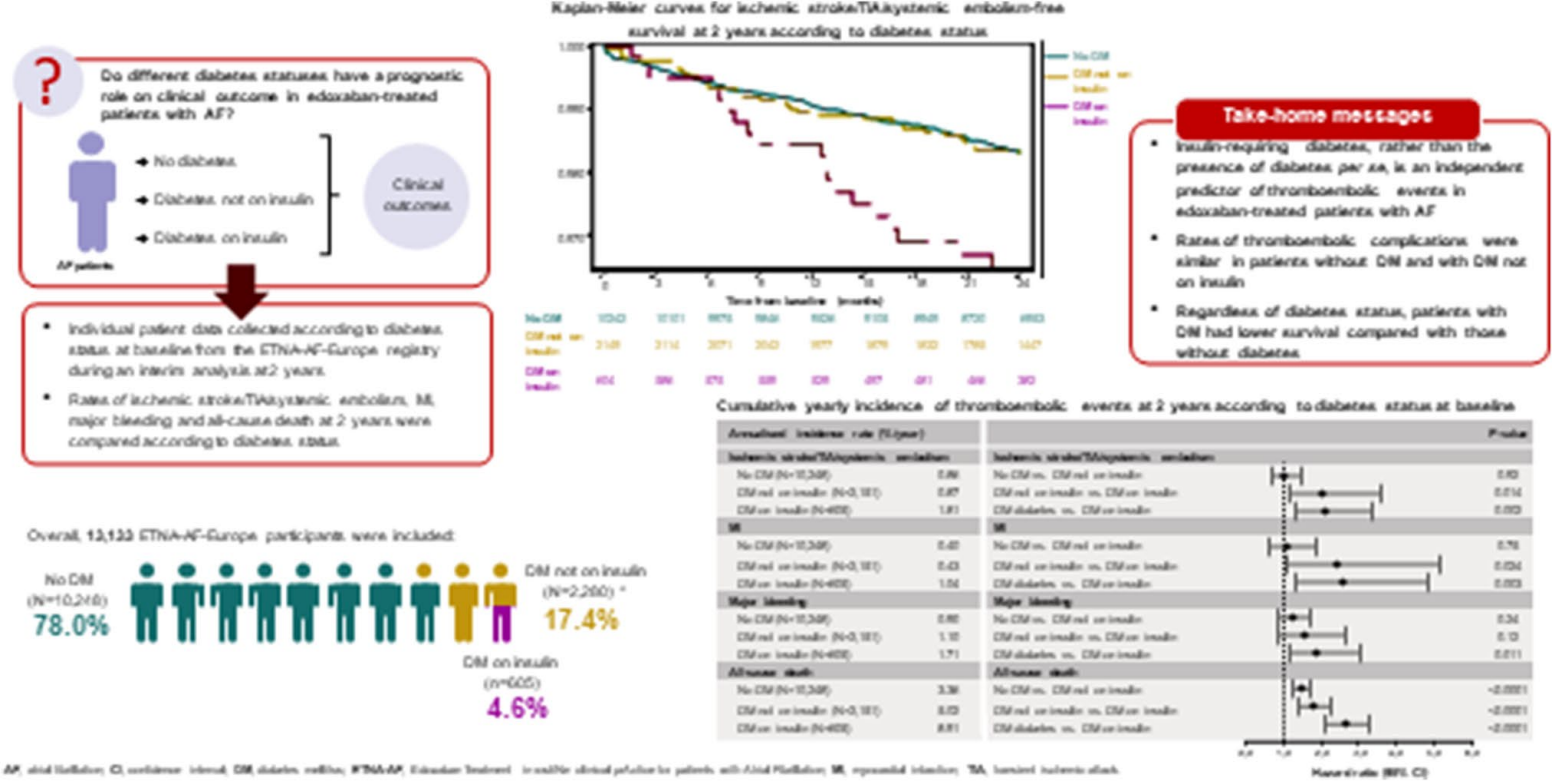

## Introduction

The presence of diabetes mellitus in patients with atrial fibrillation (AF) not receiving oral anticoagulant therapy (OAC) has been associated with a higher incidence of thromboembolic events during follow-up independently of other variables affecting cardiovascular risk [1]. Based on the above, diabetes was included in scores introduced in clinical practice for stratifying thromboembolic risk among OAC-naïve AF patients, such as the CHADS_2_ score [2] and, more recently, the CHA_2_DS_2_–VASc score [3], with the latter score being maintained in the latest guidelines [4]. This inclusion was regardless of the specific diabetes status; in particular, it did not consider whether the diabetic patient receives oral glucose-lowering drugs only or is on insulin treatment.

Recent investigations on AF patients have explored possible differences in outcome across different diabetes strata. An analysis of the prospective, real-world, Prevention of thromboembolic events–European Registry in Atrial Fibrillation (PREFER in AF) registry demonstrated, in a cohort of anticoagulated patients with AF—the large majority being on vitamin K antagonist anticoagulants (VKAs)—that the sole presence of diabetes not requiring insulin did not imply a higher risk of stroke or systemic embolism [5]; conversely, insulin-requiring diabetes mostly, if not exclusively, contributed to the overall increase of thromboembolic risk [5]. Modifications in primary or secondary hemostasis leading to a pro-thrombotic state are highly prevalent in diabetic patients with long-lasting disease receiving insulin therapy, and may explain this greater risk of thromboembolic complications, even in the background of OAC [6, 7]. Furthermore, a recent sub-analysis on anticoagulated patients with AF and diabetes enrolled in the randomized Apixaban for Reduction in Stroke and Other Thromboembolic Events in Atrial Fibrillation (ARISTOTLE) trial, highlighted a heightened cardiovascular risk, mainly related to the occurrence of myocardial infarction (MI) and cardiovascular death, largely confined to diabetes requiring insulin [8].

To date, no data are available on the prognostic role of the various diabetes statuses (diabetes on insulin vs diabetes not on insulin vs no diabetes) on clinical outcome in the setting of real-world clinical practice related to AF patients receiving non-vitamin K antagonist anticoagulants (NOACs). We have evaluated this in the prospective cohort of edoxaban-treated patients enrolled in the Edoxaban Treatment in routiNe clinical prActice for patients with Atrial Fibrillation in Europe (ETNA-AF Europe) registry.

## Methods

We accessed individual patients’ data from the ETNA-AF Europe registry (Clinicaltrials.gov: NCT02944019). ETNA-AF Europe is a prospective, observational, real-world, multinational, multicenter, post-authorization study performed at 852 sites in 10 European countries (Austria, Belgium, Germany, Ireland, Italy, The Netherlands, Portugal, Spain, Switzerland, and UK) [9–11]. ETNA-AF Europe is part of the global ETNA program, which is composed of separate, non-interventional ETNA-AF registries in Europe, East Asia (Korea and Taiwan), and Japan. The primary objective of ETNA-AF was to assess, in a real-world setting, the safety and effectiveness of edoxaban by evaluating, among the study participants, mortality, ischemic and bleeding events up to 4 years. The ETNA-AF Europe protocol was developed based on discussions with the Pharmacovigilance Risk Assessment Committee (PRAC) of the European Medicines Agency (EMA). Inclusion criteria were: age ≥ 18 years; written informed consent to participate the study; treatment with edoxaban for preventing AF-related thromboembolism, according to the drug summary of product characteristics; no participation in any interventional study. To avoid selection bias and to allow a documentation of routine clinical practice, there were no explicit exclusion criteria for the study. The registry was approved by the institutional review boards and independent Ethics Committees at all centers, in compliance with the Declaration of Helsinki and Guidelines for Good Pharmaco-epidemiological Practice (GPP). The study management was overseen by a scientific Steering Committee.

The protocol consists of a baseline evaluation at the time of patient’s enrollment, when patients’ characteristics, co-morbidities, risk factors and treatment modalities were collected. Subsequently, yearly follow-up visits were performed up to 4 years. Here we present the follow-up evaluation at 2 years according to the diabetes status at baseline. Events are as reported by the site investigator and only documented outcome events were considered as relevant, with the date of any event being after the baseline visit. Final adjudication of the main outcome measures (major bleeding, stroke, systemic embolism) was done by an independent Clinical Event Committee. All individual data were entered into an electronic case report form including various plausibility checks for the considered variables. On-site monitoring visits with complete source data verification were performed in 30% of randomly selected high recruiting centers. The registry was sponsored by Daiichi Sankyo Europe GmbH (Munich, Germany).

Diabetic patients were separately considered if they were or were not on insulin treatment and were compared with patients without diabetes. Primary study endpoint was the incidence of ischemic stroke/transient ischemic attack (TIA)/systemic embolism at 2 years according to the diabetes status (no diabetes, diabetes without insulin treatment, diabetes on insulin therapy). Stroke, TIA and systemic embolism were defined following the Effective Anticoagulation with Factor Xa Next Generation in Atrial Fibrillation–Thrombolysis in Myocardial Infarction 48 (ENGAGE AF-TIMI 48) definitions [12]: Stroke: abrupt onset of a focal neurologic deficit, generally distributed in the territory of a single brain artery (including the retinal artery), and that is not attributable to an identifiable nonvascular cause (i.e., brain tumor or trauma). The deficit must either be characterized by symptoms lasting > 24 h or cause death within 24 h of symptom onset. The occurrence of a transient focal neurologic deficit lasting ≤ 24 h identified a *TIA*. Systemic embolic event*:* abrupt episode of arterial insufficiency with clinical or radiologic documentation of arterial occlusion in the absence of other likely mechanisms (e.g., atherosclerosis, instrumentation). Venous thromboembolism and pulmonary embolism were also included in this outcome measure. Arterial embolic events involving the central nervous system (including the eye) were not considered as systemic embolism.

The following other outcome measures were considered as secondary endpoints: individual components of the primary composite endpoint; MI; major bleeding; intracranial hemorrhage; gastro-intestinal major bleeding; overall mortality; cardiovascular death. Major bleeding was defined as follows: clinically overt bleeding event, meeting at least one of the following: (a) fatal bleeding; (b) symptomatic bleeding in a critical area or organ (retroperitoneal, intracranial, intraocular, intraspinal, intra-articular, pericardial, intramuscular with compartment syndrome); (c) clinically overt bleeding event causing a fall in hemoglobin level ≥ 2.0 g/dL or a fall of hematocrit ≥ 6.0%, adjusted for transfusion [9].

Categorical variables are indicated as absolute and percentage frequencies [*n* (%)]. Continuous variables are presented as mean ± standard deviation. Kaplan Meier curves were used to present the time-to-an-event distributions. For the time-to-event analyses, the Cox proportional hazard regression models were also used in the following scenarios:

- (1) univariate models, with a categorial predictor and three categories: diabetes on insulin, diabetes not on insulin, no diabetes;
- (2) stepwise multivariate Cox model, where all variables collected at baseline, including parameters deriving from medical history, concomitant antiplatelet therapy and edoxaban dose, were added as covariates and with 3 variables (diabetes & diabetes therapy conditions) as potential predictors:
- Two levels categorial variable: "diabetes yes” vs “no diabetes";
- Three levels categorial variable: “diabetes on insulin”, “diabetes not on insulin”, “no diabetes",
- Two levels categorial variable: " no diabetes + diabetes not on insulin” vs “diabetes on insulin".
- The mathematical methodology itself objectively decided which of these 3 diabetes & diabetes therapy predictors would be in the multivariate model (or none of them), and also when enter into the model.
- (3) multivariate Cox model with a categorial predictor: diabetes on insulin, diabetes not on insulin and HbA1c value at baseline as continuous covariate. HbA1c was measured only in diabetic patients and only in 65% of them.

Hazard ratios (HR), 95% confidence intervals (CI) and corresponding *p* values are presented. All analyses are not confirmatory, but purely descriptive/exploratory; therefore, no adjustment for multiple comparison was applied. All statistical analyses were performed using SAS version 9.4.

## Results

This analysis included a total of 13,133 ETNA-AF Europe participants, whereby 2885 patients had diabetes mellitus (22.0%), 605 of whom were on insulin therapy (21.0%). Among the group of non-insulin-treated diabetic patients, 1741 were on oral antidiabetic drugs, 393 on diet, 17 on other treatments. Those diabetic patients (*N* = 129) in whom diabetes treatment was filled as unknown (*N* = 23) or not filled at all (*N* = 106) were not included in the analyses on diabetes treatment.

Regardless of insulin status, diabetic patients were older, were more frequently male, had a higher body mass index (BMI) and HAS–BLED score, as well as a higher prevalence of persistent/permanent AF, perceived frailty, systemic hypertension, heart failure, chronic obstructive pulmonary disease, peripheral artery disease and coronary heart disease vs those without diabetes (Table 1). Compared with non-diabetic patients, diabetic patients on insulin showed lower creatinine clearance and a more prevalent history of previous ischemic stroke. Compared with diabetic patients not on insulin, those receiving insulin treatment had reduced renal function, higher BMI and HbA1c levels, as well as a higher CHA_2_DS_2_–VASC score, due to a higher prevalence of heart failure, peripheral artery disease, coronary heart disease and previous ischemic stroke. A reduced 30 mg edoxaban daily dose was given in 22% of patients without diabetes, 26% of diabetic patients not on insulin and 38% of those on insulin; the prevalence of a concomitant antiplatelet treatment at baseline was 14.7, 17.2 and 17.2%, respectively.

**Table 1:** Main baseline characteristics in the study population according to diabetes status

Variable	DM on insulin *N* = 605	*p* value DM on insulin vs DM not on insulin	DM not on insulin *N* = 2151	*p* value DM not on insulin vs no DM	No DM *N* = 10,248	*p* value DM on insulin vs no DM
Age (years)	74.3 ± 8.9	0.99	74.5 ± 8.6	** < 0.0001**	73.4 ± 9.7	**0.023**
Male gender	366 (60.5%)	0.77	1,287 (59.8%)	**0.001**	5,718 (55.8%)	**0.024**
BMI (kg/m^2^)	30.5 ± 6.0	**0.004**	29.5 ± 5.4	** < 0.0001**	27.6 ± 4.9	** < 0.0001**
Type of AF						
Paroxysmal Persistent Long-standing Permanent	283 (46.8%) 145 (24.0%) 13 (2.1%) 164 (27.1%)	0.19	1023 (47.6%) 568 (26.5%) 58 (2.7%) 498 (23.2%)	** < 0.0001**	5669 (55.4%) 2440 (23.9%) 245 (2.4%) 1873 (18.3%)	** < 0.0001**
eGFR* (mL/min)						
≥ 80 50–79 30–49 15–29 < 15	178 (32.6%) 189 (34.6%) 159 (29.1%) 20 (3.7%) 0 (0.0%)	** < 0.0001**	694 (36.3%) 790 (41.4%) 369 (19.3%) 57 (3.0%) 0 (0.0%)	0.08	3216 (36.2%) 3886 (43.8%) 1562 (17.6%) 205 (2.3%) 3 (0.1%)	** < 0.0001**
CHA_2_DS_2_–VASc	4.4 ± 1.3	**0.005**	4.2 ± 1.3	** < 0.0001**	2.9 ± 1.3	** < 0.0001**
HAS–BLED	2.9 ± 1.1	0.10	2.8 ± 1.1	** < 0.0001**	2.4 ± 1.1	** < 0.0001**
Perceived frailty	96 (17.1%)	0.10	284 (14.3%)	** < 0.0001**	1012 (10.6%)	** < 0.0001**
Hypertension	548 (90.6%)	0.17	1,906 (88.6%)	** < 0.0001**	7,567 (73.8%)	** < 0.0001**
Heart failure	150 (24.8%)	**0.003**	416 (19.3%)	** < 0.0001**	1,268 (12.4%)	** < 0.0001**
COPD	83 (13.7%)	0.47	271 (12.6%)	** < 0.0001**	837 (8.2%)	** < 0.0001**
PAD	55 (9.1%)	**0.001**	117 (5.4%)	** < 0.0001**	251 (2.4%)	** < 0.0001**
CHD	231 (38.2%)	** < 0.0001**	583 (27.1%)	** < 0.0001**	1,895 (18.5%)	** < 0.0001**
Previous stroke**	55 (9.1%)	**0.009**	131 (6.1%)	0.54	589 (5.7%)	**0.001**
Previous MB	7 (1.2%)	0.54	19 (0.9%)	0.43	110 (1.1%)	0.85
Valvular disease	117 (19.3%)	0.55	393 (18.3%)	0.22	1,759 (17.2%)	0.17
HbA1c (%)	7.5 ± 1.3	** < 0.0001**	6.6 ± 1.1			
Systolic blood pressure (mmHg)	133.2 ± 17.7	0.84	134.0 ± 18.1	0.08	133.3 ± 17.9	0.48
Diastolic blood pressure (mmHg)	76.5 ± 10.1	**0.005**	78.2 ± 11.0	0.51	78.4 ± 10.8	**0.0006 **
LVEF < 40%	49 (11.3%)	0.11	126 (8.8%)	**0.027**	488 (7.1%)	**0.0011**
Antiplatelet therapy	104 (17.2%)	0.98	369 (17.2%)	** 0.004**	1,506 (14.7%)	0.09
Edoxaban dose at baseline						
60 mg 30 mg	378 (62.5%) 227 (37.5%)	** < 0.0001**	1588 (73.8%) 563 (26.2%)	** < 0.0001**	7972 (77.8%) 2276 (22.2%)	** < 0.0001**

Values are expressed as *n* (%) or mean ± standard deviation

Significant *p* values are reported in bold

*AF* atrial fibrillation, *BMI* body mass index, *CHD* coronary heart disease, *COPD* chronic obstructive pulmonary disease, *DM* diabetes mellitus, *eGFR* estimated glomerular filtration rate, *MB* major bleeding, *LVEF* left ventricular ejection fraction, *PAD* peripheral artery disease

*By Cockroft–Gault equation

**Ischemic stroke only. A total of 94 patients had incomplete baseline data on characteristics listed in the first column and were not included in the table. HbA1c values were available in 1869 patients with diabetes

**Fig. 1:**
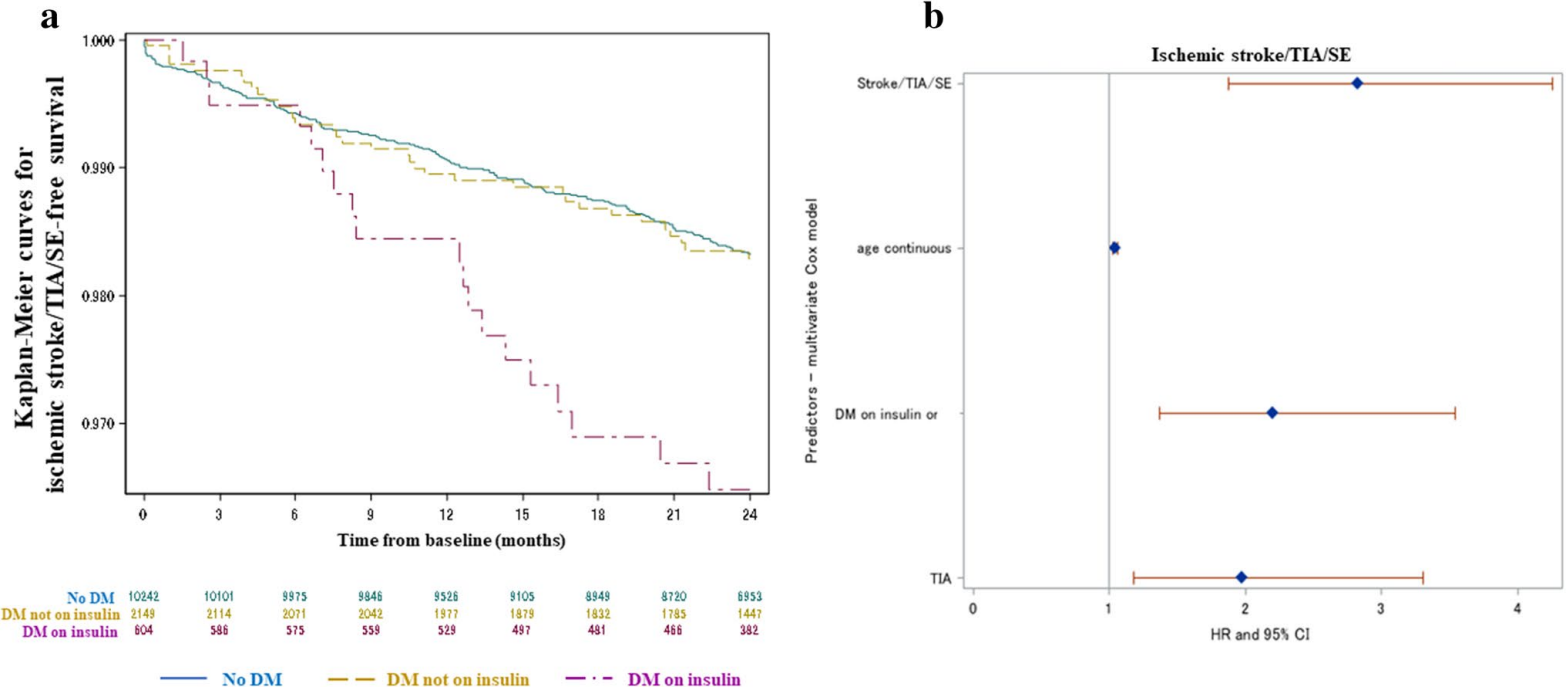
**a** Kaplan–Meier curves for ischemic stroke/TIA/SE-free survival at 2 years according to diabetes status. **b** Results of multivariate Cox model for cumulative incidence of ischemic stroke/TIA/SE. Significant predictors were DM on insulin, age and previous stroke/TIA/SE *CI *  confidence interval, *DM * diabetes mellitus, *HR *  hazard ratio, *SE*   systemic embolism, *TIA*   transient ischemic attack

The distribution of adverse events across different diabetes strata is reported in Table 2. The cumulative incidence of thromboembolic events, including ischemic stroke/TIA/systemic embolism at 2 years, was similar in patients with non-insulin-treated diabetes and without diabetes (0.87%/year vs 0.86%/year; HR 1.02, 95% CI 0.70–1.47, *p* = 0.92) (Fig. 1). Insulin-requiring diabetes was associated with a higher risk of ischemic stroke/TIA/systemic embolism (1.81%/year) vs both no diabetes (HR 2.10, 95% CI 1.31–3.38; *p* = 0.002) and diabetes not on insulin (HR 2.06, 95% CI 1.18–3.62; *p* = 0.014). After adjustment for potential confounders based on the multivariate Cox model, the correlation between diabetes on insulin and a higher occurrence of thromboembolic events remained significant (stepwise selection adjusted HR 2.20, 95% CI 1.37–3.54, *p* = 0.0011 vs no diabetes + diabetes not on insulin; HbA1c adjusted HR 2.13, 95% CI 1.12–4.05, *p* = 0.021 vs no diabetes) (Fig. 1). Consistent results were found for individual components of the composite measure of thromboembolic events. Stroke incidence was 0.58%/year in patients without diabetes, 0.64%/year in diabetic patients not receiving insulin (HR 1.11, 95% CI 0.72–1.71, *p* = 0.64 vs no diabetes) and 1.80%/years in diabetic patients receiving insulin (HR 3.11, 95% CI 1.91–5.07, *p* < 0.0001 vs no diabetes).

**Table 2:** Crude rates of adverse events at 2-year follow-up

Variable	DM on insulin	HR, 95% CI and *p* value DM on insulin vs DM not on insulin	DM not on insulin	HR, 95% CI and *p* value DM not on insulin vs no DM	No DM	HR, 95% CI and *p* value DM on insulin vs no DM
Ischemic stroke, TIA, SE	19 (1.81)	2.06, 1.18–3.62. *p* = **0.014**	34 (0.87)	1.02, 0.70–1.47, *p* = 0.92	161 (0.86)	2.10, 1.31–3.38, *p* =** 0.002**
Any stroke	19 (1.80)	2.80, 1.54–5.08, *p* =** 0.001**	25 (0.64)	1.11, 0.72–1.71, *p* = 0.64	109 (0.58)	3.11, 1.91–5.07, *p* <** 0.0001**
SE	0	–	4 (0.10)	2.15, 0.66–6.97, *p* = 0.20	9 (0.05)	
Myocardial infarction	11 (1.04)	2.40, 1.12–5.12, *p* =** 0.024**	17 (0.43)	1.09, 0.64–1.85, *p* = 0.75	75 (0.40)	2.58, 1.37–4.86, *p* =** 0.003**
Major bleeding	18 (1.71)	1.55, 0.89–2.68, *p* = 0.12	43 (1.10)	1.22, 0.87–1.71, *p* = 0.24	170 (0.90)	1.88, 1.16–3.06, *p* =** 0.011**
Intracranial hemorrhage	6 (0.56)	3.14, 1.06–9.35, *p* =** 0.040**	7 (0.18)	0.97, 0.43–2.17, *p* = 0.93	35 (0.18)	3.04, 1.28–7.22, *p* =** 0.012**
Major gastro-intestinal bleeding	5 (0.47)	0.77, 0.29–2.01, *p* = 0.59	24 (0.61)	1.68, 1.06–2.67, *p* =** 0.029**	69 (0.36)	1.29, 0.52–3.19, *p* = 0.59
All-cause death	95 (8.91)	1.78, 1.39–2.27, *p* <** 0.0001**	197 (5.02)	1.50, 1.27–1.75, *p* <** 0.0001**	637 (3.36)	2.66, 2.14–3.30, *p* <** 0.0001**
Cardiovascular death	49 (4.60)	1.68, 1.20–2.36, *p* =** 0.003**	107 (2.72)	1.45, 1.17–1.80, *p* =** 0.001**	357 (1.88)	2.44, 1.81–3.29, *p* <** 0.0001**

Events are reported as *n* (%/year)

Significant *p* values are reported in bold

*CI* confidence interval, *DM* diabetes mellitus, *HR*  hazard ratio, *SE* systemic embolism, *TIA* transient ischemic attack

**Fig. 2:**
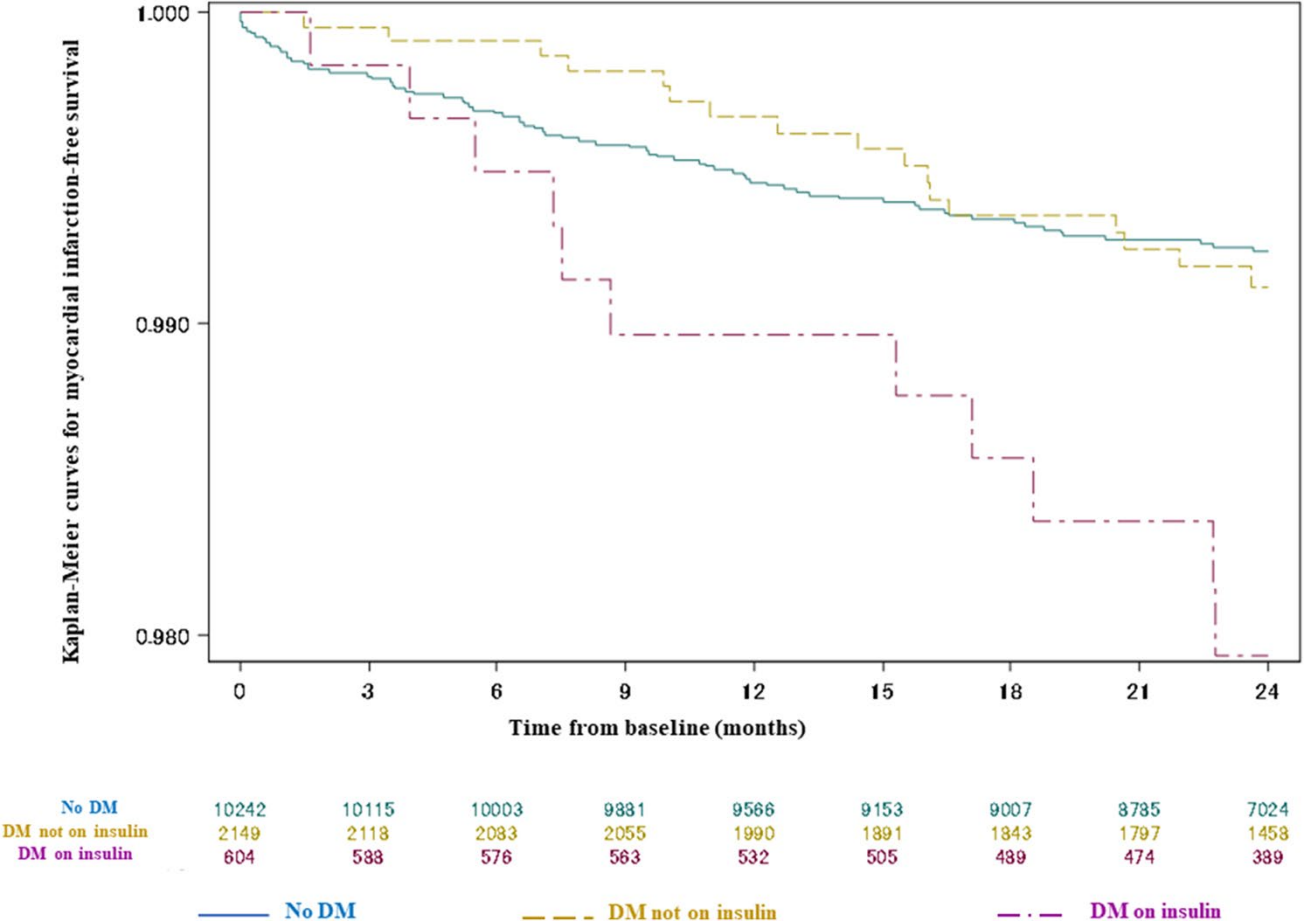
Kaplan–Meier curves for myocardial infarction-free survival according to diabetes status. *DM*   diabetes mellitus

The rates of MI during follow-up were comparable in patients with diabetes not on insulin and without diabetes (0.43%/year vs 0.40%/year; HR 1.09, 95% CI 0.64–1.85, *p* = 0.75) (Fig. 2). Insulin-requiring diabetes had a higher risk of MI (1.04%/years) vs both no diabetes (HR 2.58, 95% CI 1.37–4.86, *p* = 0.003) and non-insulin-requiring diabetes (HR 2.40, 95% CI 1.12–5.12, *p* = 0.024) (Table 2). However, at multivariate analysis the presence of insulin-treated diabetes was here not an independent predictor of developing an MI within 2 years (mathematical selection did not identify any of 3 diabetes and diabetes therapy potential predictors into the stepwise selection multivariate Cox model as statistically significant; HbA1c-adjusted HR was 2.10, 95% CI 0.78–5.66, *p* = 0.14 vs diabetes not on insulin).

**Fig. 3:**
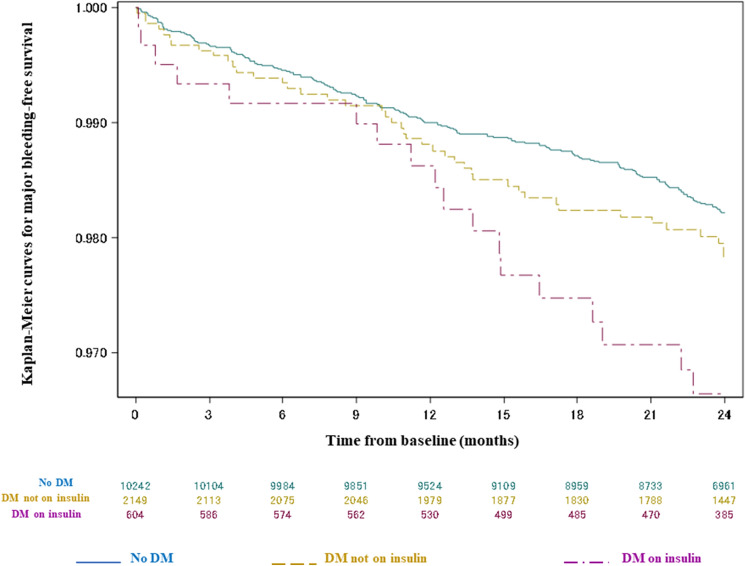
Kaplan–Meier curves for major bleeding-free survival at 2 years according to diabetes status. *DM*   diabetes mellitus

The occurrence of major bleeding was similar in patients with diabetes not requiring insulin and in non-diabetic patients (1.10%/year vs 0.90%/year; HR 1.22, 95% CI 0.87–1.71, *p* = 0.24) (Fig. 3). Diabetic patients on insulin showed a significantly higher rate of major bleeding (1.71%/year) vs non-diabetic patients (HR 1.88, 95% CI 1.16–3.06, *p* = 0.011), but this was not proved as statistically significant vs diabetic patients without insulin treatment (HR 1.55, 95% CI 0.89–2.68, *p* = 0.12) (Table 2). Mathematical selection did not identify any of 3 diabetes and diabetes therapy potential predictors into the stepwise selection multivariate Cox model as statistically significant. HbA1c-adjusted HR for the association between diabetes on insulin and a higher risk of major bleeding was 1.81 (95% CI 0.95–3.44, *p* = 0.071 vs diabetes not on insulin). Rates of intracranial hemorrhage were 0.18%/year in non-diabetic patients, 0.18%/year in diabetic patients without insulin therapy (HR 0.97, 95% CI 0.43–2.17, *p* = 0.93 vs no diabetes) and 0.56%/year in diabetic patients on insulin (HR 3.04, 95% CI 1.28–7.22, *p* = 0.012 vs no diabetes) (Table 2).

**Fig. 4:**
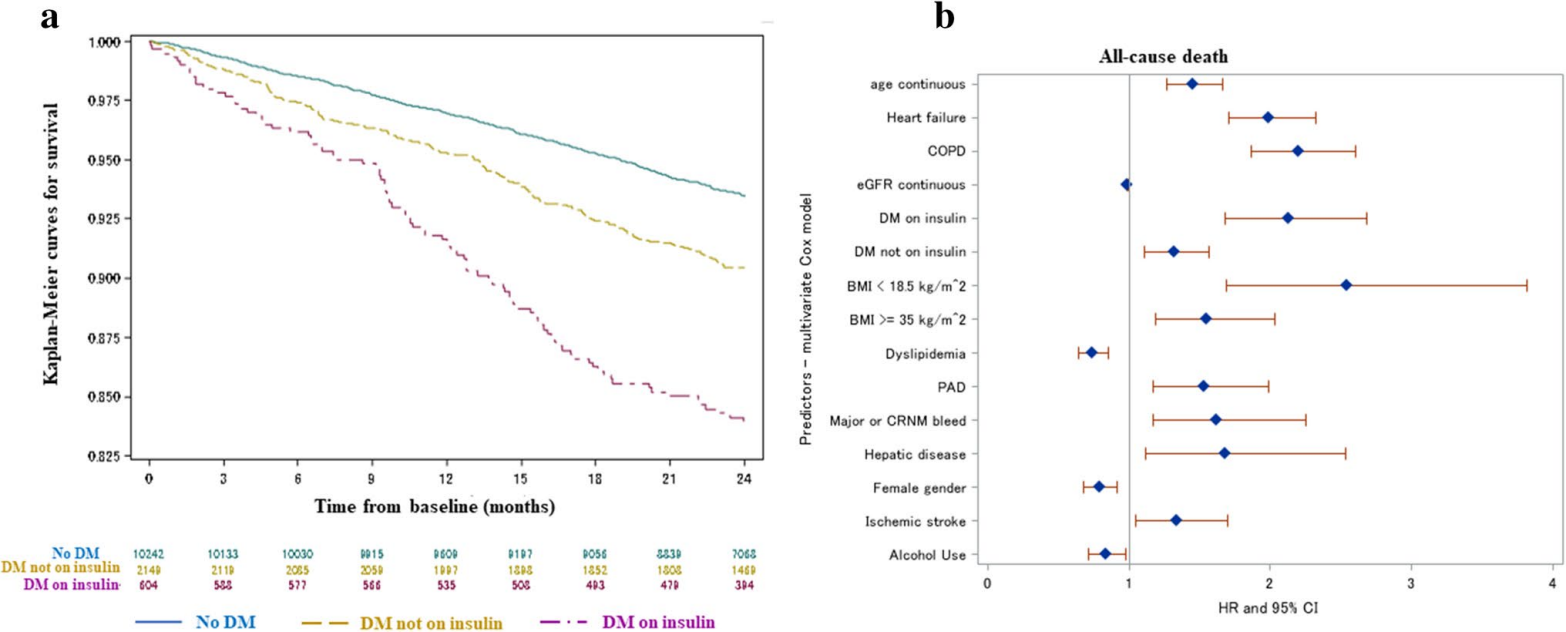
**a** Kaplan–Meier curves for survival at 2 years according to diabetes status. **b** Results of multivariate Cox model for all-cause death. *BMI*   body mass index, *CI *  confidence interval, *COPD*   chronic obstructive pulmonary disease, *CRNM *  clinically relevant non-major bleeding, *DM*   diabetes mellitus, *eGFR*   estimated glomerular filtration rate, *HR* hazard ratio, *PAD*   peripheral artery disease

Overall mortality was 3.36%/year in patients without diabetes, 5.02%/year in those with diabetes without insulin therapy (HR 1.50, 95% CI 1.27–1.75, *p* < 0.0001 vs no diabetes) and 8.91%/year in insulin-treated patients (HR 2.66, 95% CI 2.14–3.30, *p* < 0.0001 vs no diabetes) (Fig. 4). HR for all-cause death in diabetic patients with vs without insulin therapy was 1.78 (95% CI 1.39–2.27, *p* < 0.0001) (Table 2). After adjustment for potential confounders based on the multivariate Cox model, the presence of diabetes remained associated with lower survival, regardless of the need for insulin therapy (Fig. 4): stepwise selection adjusted HR for diabetes on insulin vs no diabetes was 2.13 (95% CI 1.68–2.68, *p* < 0.0001), for diabetes not on insulin vs no diabetes was HR 1.32 (95% CI 1.11–1.57, *p* = 0.0015). Notably, the adjusted HR was significant also in the comparison between diabetic patients receiving insulin vs those not receiving insulin, with a 61% higher mortality in the former via stepwise selection adjusted HR of 1.61 (95% CI 1.25–2.09, *p* = 0.0003), and an 83% higher mortality via HbA1c-adjusted HR of 1.83 (95% CI 1.35–2.49, *p* = 0.0001). Consistent results with those observed for all-cause death were found in the sensitivity analyses on cardiovascular mortality.

## Discussion

This real-world study highlights an important heterogeneity in diabetes as a risk factor in AF patients receiving a NOAC. In fact, diabetes requiring insulin therapy, rather than the presence of diabetes per se, appeared to be an independent factor affecting the occurrence of thromboembolic complications during follow-up. Furthermore, the diabetic condition was characterized by a lower survival at 2 years vs no diabetes regardless of the need for insulin use, but all-cause mortality in the subgroup with diabetes on insulin was higher compared with diabetes not on insulin.

Diabetes increases the risk of developing AF, because of atrial inflammation, oxidative stress, electrical/structural atrial remodeling and changes in the autonomic response [13–15]. Thus, the prevalence of diabetes among AF patients is high, ranging approximately from 25 to 40% in contemporary trials [16]. Phase III randomized trials on AF confirmed an improved outcome with NOACs vs VKAs also in the subgroup with diabetes mellitus [17]. This analysis from the ETNA-AF Europe registry provides reassuring results regarding the efficacy and safety of edoxaban among diabetic patients with AF also in a real-world setting, with annual rates of adverse events even lower than in the diabetic population of the ENGAGE AF–TIMI 48 trial on the higher dose edoxaban regimen (stroke or systemic embolism: 0.98% vs 1.42%; major bleeding: 1.23% vs 3.2%) [12]. The above-mentioned 0.98% yearly incidence of stroke or systemic embolism in ETNA-AF Europe was also lower than that observed in the diabetic subset of the PREFER in AF registry (2.6%), where patients were predominantly treated with VKAs [5].

The differential impact of a different diabetes status on outcome in AF is a matter of recent clinical investigation. In the present study, as compared with diabetic patients not on insulin, those receiving insulin had higher CHA_2_DS_2_–VASC score and more frequently criteria for edoxaban dose reduction (mainly due to a reduced creatinine clearance). After adjustment for potential confounders, including co-morbidities, glycemic control and concomitant treatments, we found that diabetes on insulin was associated with a 2.2-fold higher risk of ischemic stroke/TIA/systemic embolism vs no diabetes or diabetes not on insulin. This corroborates and strengthens the similar relative increase of thromboembolic complications observed in the insulin-treated diabetic subpopulation of the PREFER in AF registry [5]. Thus, a comprehensive interpretation of both studies supports that the residual risk of thromboembolic events among AF patients with diabetes requiring insulin therapy is not insignificant in a real-world setting, even in the background of chronic OAC and regardless of the OAC type (VKAs or NOACs). A clustering of concomitant diseases and risk factors likely may also contribute to this increased risk in diabetic patients on insulin and insulin use is likely to be a proxy for difficult glycemic control and/or longer duration of diabetes in patients with atrial fibrillation. Furthermore, in diabetic patients a more pronounced platelet reactivity and a hypercoagulable milieu have been described, both being particularly evident in those with long lasting disease receiving insulin treatment [14, 18–20]. A high inflammatory status, oxidative stress and endothelial dysfunction may trigger these effects on platelets and coagulative mechanisms [6, 21]. However, increased levels of advanced glycosylation end-products or direct effects of exogenous insulin, with pathologically high levels of insulin in a background of insulin resistance, might also have a role [22–24]. Notably, our results are consistent with recent reports indicating a clustering of adverse events in diabetic patients on insulin with heart failure at either reduced or preserved ejection fraction [25, 26].

The previous PREFER in AF analysis had also shown the much less obvious finding that the occurrence of thromboembolic events in diabetic patients not receiving insulin is similar to that of patients without diabetes [5]. However, the possibility that here a difference in such comparison could not be demonstrated because of a potential type II statistical error, due to a low sample size (*n* = 6412) and a follow-up of only 1 year, exists. Those PREFER-in AF results have been now by-and-large confirmed on the larger population of ETNA-AF Europe. Thus, robust evidence now indicates that, in the setting of AF, long-term oral anticoagulation appears to equalize the risk of thromboembolic complications of diabetic patients without insulin treatment to that of non-diabetics. Notably, recent data on AF demonstrated that, despite anticoagulant therapy, thrombin generation is increased in diabetic patients receiving insulin vs those without diabetes or with diabetes on oral antidiabetic drugs, with no difference between these latter two conditions [7]. Thus, an imbalanced thrombin generation might be a predominant contributor to the excess thromboembolic risk in patients with insulin-requiring diabetes, whereas a balanced thrombin generation could explain the comparable thromboembolic risk in those with diabetes not on insulin and no diabetes.

Experimental and clinical data on the potential atherogenic effects of insulin are controversial [27, 28]. Interestingly, in the sub-analysis of the ARISTOTLE trial based on the diabetes status, only insulin-treated diabetes was a predictor of future MIs (HR 2.34) compared with no diabetes [8]. This was regardless of the OAC type (warfarin or apixaban). In the present investigation based on real-world clinical practice, we observed that insulin-requiring diabetes was associated with a very similar 2.35-fold elevation of MI. Due to the low event rates, such association was not significant at multivariate analysis. However, we confirmed the similar rates of MI in patients with diabetes without insulin therapy and in those without diabetes.

We also found a stepwise increase in all-cause death at 2 years across different diabetes strata. In particular, the adjusted excess mortality vs patients without diabetes was 1.3-fold in diabetic patients not receiving insulin and 2.1-fold in those on insulin. Of note, the excess stroke/TIA/systemic embolism in patients with insulin-requiring diabetes was paralleled by a consistent increase in all-cause death and cardiovascular death. Our findings also show that: regardless of the need for insulin treatment, AF patients with diabetes feature a significantly lower survival compared with those without diabetes; diabetic patients on insulin have an 83% higher all-cause mortality vs diabetic patients not on insulin. These results are in line with previous evidence that an effective OAC in AF patients compresses the rates of thromboembolic complications and MI; as a consequence, the risk of cardiovascular death exceeds the risk of stroke, systemic embolism and MI, with the cardiovascular mortality excess being driven by non-thrombotic events, such as heart failure or lethal arrhythmias [29]. The present ETNA-AF Europe analysis supports that the diabetes condition, regardless of insulin therapy, may also be associated with the development of the above-mentioned non-thrombotic causes of death, beside with causes of non-cardiovascular mortality.

A previous report from phase III randomized trials in AF, where data on patients receiving NOACs and warfarin were pooled together, showed a higher occurrence of bleeding complications in the subgroup with vs without diabetes [17]. In the present study, the rates of major bleeding while on edoxaban treatment were comparable in diabetic patients not on insulin and in non-diabetic patients. A numerical increase of major bleeding in diabetes was confined to the subset with insulin treatment; however, the latter condition was not an independent predictor of a poorer haemorrhagic outcome due to the low event rates, with a p value at multivariate analysis being very close to the statistical significance. Results on intracranial haemorrhage and major gastro-intestinal bleeding were consistent. Mechanisms for the association between insulin-requiring diabetes and a higher bleeding risk are unclear, with a possible role of micro-angiopathy in patients suffering from long-lasting disease and a hyperglycemia-related vascular leakage being here invoked [30]. Notably, with non-diabetic patients as reference, recent data on patients with acute coronary syndrome demonstrated a similar risk of the net composite endpoint, including cardiovascular death, MI, stroke and major bleeding at 1 year, in diabetic patients not receiving insulin and a significantly higher risk in those on insulin [31]. Such increase in the latter was driven by both ischemic cardiovascular events and major bleeding complications.

The present investigation has strengths in being a prospective analysis on a large real-world population of AF patients treated with a specific, newer anticoagulant agent undergoing a detailed baseline assessment and planned follow-up evaluations up to 2 years, with external event adjudication and a source data verification in 30% of sites. Limitations are inherent to all observational investigations. In particular, a selection bias and residual confounding cannot be excluded. Our results refer to patients with type 2 diabetes; in this regard it is possible that insulin provision in type 1 diabetes, in the absence of insulin resistance, is not associated with a poorer prognosis. We had no data to adjust the association between type of diabetes and adverse events for diabetes duration. However, we stigmatize that, in the previous PREFER in AF analysis, the predictive role of insulin-requiring diabetes on outcomes was independent of diabetes duration. We adjusted results by multivariate analysis; however, it per se is not accounting for all biological variations. In particular, results were adjusted for glycemic control, but this was not feasible in approximately one third of patients with diabetes, and adjustment for over time variation of glycemic control was not possible. Furthermore, we did not correct the events incidence for other sources of heterogeneity, such as microvascular complications, different types of non-insulin medications and the association of insulin plus oral antidiabetic drugs, as these variables were not collected. In particular, it was not possible to correct results for the use of oral antidiabetic agents with proven beneficial cardiovascular effects, such as sodium glucose transporter-2 inhibitors and glucagon-like peptide-1 agonists, which were very marginally used at the time of ETNA-AF initiation. Thus, it is unlikely that an imbalance in the use of these drugs with proven cardiovascular benefit might explain the differences among our groups. Possible imbalances, for which we did not adjust in the absence of specific information, could never, however, explain the absence of excess risk in diabetic patients not on insulin vs non-diabetic patients for stroke/TIA/systemic embolism, MI and bleeding, and, therefore, cannot be considered a substantial limitation of the present report. Indeed, the choice of treatment of diabetic patients, including insulin use, was in various cases performed by the treating general physician; as there are no reliable criteria for the decision for or against the use of insulin, this may pose a certain bias. Finally, we were not able to explore the relationship between diabetes status and the incidence of coronary revascularization in the current era of newer drug-eluting coronary stents [32].

In conclusions, the findings of this study indicate a quite dichotomous behavior of anticoagulated AF population with diabetes according to the use or lack of use of insulin. Our data indicate a segregation of a higher cardiovascular risk for non-fatal events, with potential impact on survival, only to AF patients with insulin-requiring diabetes. Altogether, the evidence on this segregation is now based on an overall population of over 37,000 patients from three independent cohorts (PREFER in AF registry; ARISTOTLE trial; ETNA-AF Europe registry). These results may have an application in the assessment of the residual thromboembolic risk in the background of OAC among patients with AF and concomitant diabetes. Our findings might have also therapeutic implications, but, as association does not prove causation, they warrant further investigation in dedicated prospective, intervention studies.
